# Trajectories of physical function prior to death and brain neuropathology in a community-based cohort: the act study

**DOI:** 10.1186/s12877-017-0637-7

**Published:** 2017-11-02

**Authors:** Andrea Z. LaCroix, Rebecca A. Hubbard, Shelly L. Gray, Melissa L. Anderson, Paul K. Crane, Joshua A. Sonnen, Oleg Zaslavsky, Eric B. Larson

**Affiliations:** 1 Division of Epidemiology, Department of Family Medicine and Public Health, University of California San Diego, 9500 Gilman Drive, La Jolla, CA 92093 USA; 2 Kaiser Permanente Washington Health Research Institute, 1730 Minor Avenue, Suite 1600, Seattle, WA 98101 USA; 3 Department of Biostatistics, Epidemiology, and Informatics, University of Pennsylvania, 423 Guardian Drive, Philadelphia, PA 19104 USA; 4 Schools of Medicine and Pharmacy, University of Washington, 1959 NE Pacific Street, Seattle, WA 98195 USA; 5 Department of Pathology, University of Utah, 15 North Medical Drive East, Suite 1100, Salt Lake City, UT 84112 USA; 6 School of Nursing, University of Washington, 1959 NE Pacific Avenue, Seattle, WA 98195 USA

**Keywords:** Physical function, Functional decline, Brain neuropathology, Alzheimer’s disease, Vascular dementia

## Abstract

**Background:**

Mechanisms linking cognitive and physical functioning in older adults are unclear. We sought to determine whether brain pathological changes relate to the level or rate of physical performance decline.

**Methods:**

This study analyzed data from 305 participants in the autopsy subcohort of the prospective Adult Changes in Thought (ACT) study. Participants were aged 65+ and free of dementia at enrollment. Physical performance was measured at baseline and every two years using the Short Physical Performance Battery (SPPB). Data from 3174 ACT participants with ≥2 SPPB measurements were used to estimate two physical function measures: 1) rate of SPPB decline defined by intercept and slope; and 2) estimated SPPB 5 years prior to death. Neuropathology findings at autopsy included neurofibrillary tangles (Braak stage), neuritic plaques (CERAD level), presence of amyloid angiopathy, microinfarcts, cystic infarcts, and Lewy bodies. Associations (adjusted for sex, age, body mass index and education) between dichotomized neuropathologic outcomes and SPPB measures were estimated using modified Poisson regression with inverse probability weights (IPW) estimated via Generalized Estimating Equations (GEE). Relative risks for the 20^th^, 40^th^, and 60^th^ percentiles (lowest levels and highest rates of decline) relative to the 80th percentile (highest level and lowest rate of decline) were calculated.

**Results:**

Decedents with the least vs. most SPPB decline (slope > 75^th^ vs. < 25^th^ percentiles) had higher SPPB scores, and were more likely to be male, older, have higher education, and exercise regularly at baseline. No significant associations were observed between neuropathology findings and rate of SPPB decline. Lower predicted SPPB scores 5 years prior to death were associated with higher risk of microinfarcts (RR = 3.08, 95% confidence interval (CI) 0.93–1.07 for the 20^th^ vs. 80^th^ percentiles of SPPB) and significantly higher risk of cystic infarcts (RR = 2.72, 95% CI 1.45–5.57 for 20^th^ vs. 80^th^ percentiles of SPPB).

**Conclusion:**

Cystic infarcts and microinfarcts, but not neuropathology findings of Alzheimer’s disease, were related to physical performance levels five years before death. No pathology findings were associated with rates of physical performance decline. Physical function levels in the years prior to death may be affected by vascular brain pathologies.

## Background

The US population of adults aged 65 and older has multiplied over 12-fold since 1900 reaching 40.3 million in 2010, and is projected to more than double again to 83.7 million by 2050 [1]. Mortality rates continue to decline among those aged 65 and older, but older Americans place a higher value on remaining in good health and being able to take care of themselves than they do on longevity [2]. The prevalence of disability in older Americans is estimated at 38% [1] including physical, cognitive, sensory, and self-care disabilities. Thus, identifying the causes of functional declines and effective interventions to reverse these losses is extremely salient for older adults, and a major societal concern.

Evidence emerging over the last decade suggests that cognitive and physical function are linked [3] but the temporal nature of declines in each domain and underlying mechanisms are poorly understood. Older adults rely on more regions of their brain for movement than younger people [4]. Lower grip strength and gait speed have been associated with markers of brain aging including brain atrophy and white matter hyperintensities [5]. However, the role of brain pathology in driving declines in physical function is virtually unknown. Frailty is a composite construct including weakness, slowness, low physical activity, weight loss and fatigue. When frailty is measured shortly before death it has been associated with pathology related to Alzheimer’s Disease [6]. Moreover, the rate of change in frailty over a 6-year period has been associated with multiple brain neuropathologies including macroinfarcts, Alzheimer’s Disease (AD) and Lewy body pathology, and nigral neuronal loss [7]. However, other studies of frailty and incident dementia have shown associations between frailty and non-AD but not AD dementia [8–10]. In the Adults Changes in Thought (ACT) study, physical function [11] and frailty [10] both predicted onset of dementia, but it is not known whether these associations were related to brain neuropathologies indicative of AD or non-AD dementia or both.

The objective of this analysis was to test the hypothesis that trajectories of physical function and physical function prior to death were associated with neuropathologic findings typical of AD or vascular dementia among participants in the ACT study who died and consented to autopsy.

## Methods

### Study population

ACT recruits community-dwelling, non-demented adults age 65 and older from among Group Health (GH) members living in or near Seattle, Washington. The original cohort of 2581 people was enrolled between 1994 and 6 and an expansion cohort (*n* = 811) was enrolled between 2000 and 2002. In 2004, the study began ongoing enrollment to replace people who die or drop out. In all phases, potential participants were randomly selected from eligible GH members. A total of 4415 participants had been enrolled at the time of this analysis.

**Fig. 1:**
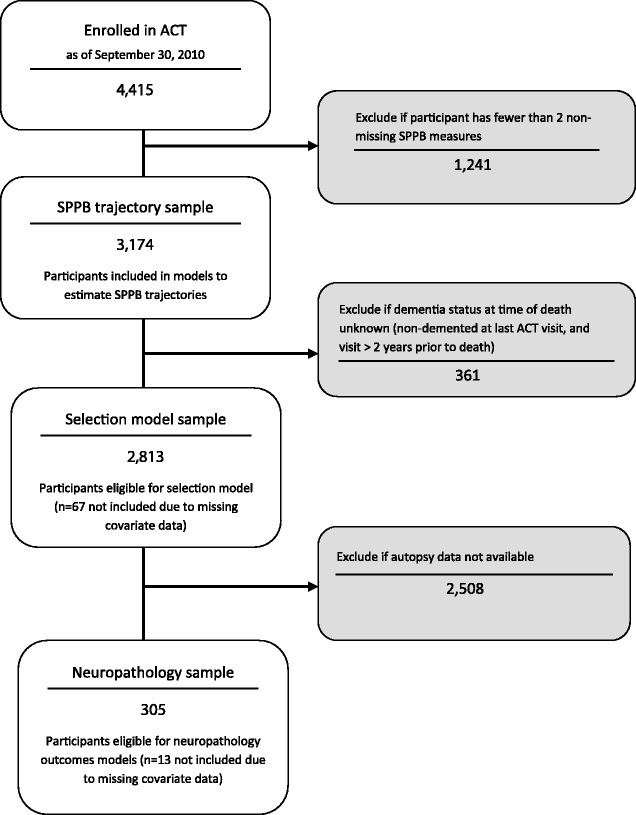
Flow diagram of ACT participants and autopsy subcohort with neuropathological measurements

Models used to characterize physical performance trajectories are based on the subset of 3174 individuals with at least two Short Physical Performance Battery [12, 13] measurements (Fig. 1). Dementia status at the time of death is an important predictor for selection into the autopsy cohort (individuals who died and came to autopsy), therefore selection models used to estimate and adjust for the probability of inclusion in the autopsy sample exclude people whose dementia status at the time of death was not known. The subset of 305 individuals who died and came to autopsy were included in analyses of neuropathologic outcomes (Fig. 1).

### Physical performance

Physical performance was measured at baseline, and every two years during study follow-up. The ACT SPPB protocol was based on a validated, composite measure of objective physical performance that included: the ability to stand with feet together in the side-by-side, semi-tandem, and tandem positions (i.e. balance), 10 ft timed walk, and the time to rise from a chair and return to the seated position 5 times. Each item (balance, gait speed, and chair stands) is scored on a 0–4 scale, and summed to define a composite score with a range of 0–12, with higher scores indicating higher function [12, 13]. We used linear mixed effects models to estimate subject-specific slopes and intercepts, which were used to describe SPPB trajectories. We estimated trajectories of SPPB for all ACT participants with at least two non-missing SPPB scores, rather than limiting to the ubsample with neuropathology data, so that the distribution of slopes and intercepts estimated by the random effects model would reflect the distribution among the population of older adults more generally. For deceased ACT participants for whom neuropathology data were available, we computed the predicted SPPB five years prior to death based on the participant’s age at death and the slope and intercept estimated from his or her SPPB data. The estimated subject-specific slopes and SPPB five years prior to death were the primary exposure measures.

### Outcomes

Neuropathologic assessments were performed and dichotomized at the level previously associated with dementia [14, 15] by board certified neuropathologists at the Alzheimer ‘s disease Research Center, Neuropathology Core in the Department of Pathology at the University of Washington. Changes indicative of AD (neuritic plaques assessed by Consortium to Establish a Registry for Alzheimer’s Disease staging criteria [16, 17], neurofibrillary tangles assessed by Braak staging [18, 19], and amyloid angiopathy [20]), cerebrovascular disease (cerebral microinfarcts, macroscopic infarcts, and atherosclerosis), Lewy bodies, and hippocampal sclerosis [21, 22] were assessed. Details have been published ([23] see eMethods 5). Briefly, for Alzheimer pathology, functional dichotomization identified neuritic plaques by the presence of six or more neuritic plaques identified by Bielschowsky sliver staining in a single high powered field of middle frontal gyrus or inferior parietal lobule, neurofibrillary tangles pathology was dichotomized by the presence of tangles in all field of the hippocampus and entorhinal cortex as well as association cortex by tau immunohistochemistry, and amyloid angiopathy was dichotomized by the presence of circumferential amyloid staining by Congo red histochemistry in a screening section of cortex. For vascular pathology, cystic infarcts were identified at the time of gross examination and confirmed as chronic infarcts by histology while microinfarcts pathology was dichotomized as positive if two or more were identified at the time of histologic exam as chronic infarcts but not identified as chronic infarcts. Hippocampal sclerosis was defined present is there was complete loss of neurons within the CA1 field of the hippocampus with or without involvement of the subiculum. Lewy bodies were classified as present when identified by immunohistochemistry for alpha-synuclein in a section of the frontal cortex. Corticol Lewy body indices were obtained but were too infrequent to be included in the analysis. No measurements of long-tract white matter disease are currently available.

### Covariates

Demographic characteristics including age, sex, educational attainment, and study cohort were collected at baseline. Study participants reported smoking status, and regular exercise (defined as 15 min or more at least 3 times per week), and body mass index (BMI) was calculated based on measured height and weight at baseline and each follow-up visit. Grip strength was measured as part of the physical performance assessment at baseline and study follow-up visits. Methods for diagnosing dementia in ACT have been previously reported [24]. Briefly, participants complete cognitive screening at biennial study visits, and those with low scores are referred for further evaluation with a clinical examination and a neuropsychological battery. All data are then reviewed by a consensus committee that assigns dementia diagnoses based on the Diagnostic and Statistical Manual of Mental Disorders, 4^th^ Edition (DSM-IV).

### Statistical analysis

Descriptive counts and percentages for covariates were computed for dichotomized neuropathologic outcomes by quartiles of estimated subject-specific SPPB slopes. The quartiles were defined based on the distribution of SPPB trajectories in the autopsy cohort.

Analyses of the association between dichotomized neuropathologic outcomes and characteristics of SPPB trajectories were conducted using modified Poisson regression with inverse probability weights (IPW) estimated via Generalized Estimating Equations (GEE). Modified Poisson regression allowed us to estimate relative risks of more severe neuropathology outcomes rather than odds ratios, which are more common but difficult to interpret in the case of binary outcomes [25]. IPW were used to account for the selection mechanisms resulting in inclusion in our autopsy cohort. By including these weights we were able to estimate associations between neuropathology and SPPB trajectories that are generalizable to the ACT cohort as a whole. Selection models used to compute the weights included terms for sex, race, education, ACT cohort, and characteristics measured at last study follow-up visit including dementia status, exercise, smoking, and BMI. Neuropathology outcome models were adjusted for sex, education, and age and BMI measured at the last study follow-up visit. We obtained confidence intervals and *p*-values for neuropathology outcome models via bootstrap sampling to account for uncertainty in the selection model as well as the neuropathology outcome model. We used a bias-corrected and accelerated bootstrap procedure [26] with 1000 samples from the bootstrap distribution to estimate the sampling distribution of model parameters.

Each neuropathology outcome was evaluated in separate models testing each of two predictors describing the SPPB trajectory: the subject-specific slope of SPPB and the predicted SPPB five years prior to death. Natural cubic splines with two degrees of freedom were used to describe associations between these predictors and the log-risk of an adverse neuropathologic outcome. Splines were used to model these associations to avoid imposing assumptions about the functional form of relationships between characteristics of the SPPB trajectory and risk of adverse neuropathologic outcomes. Relative risks (RR) were computed for the 20^th^, 40^th^, and 60^th^ percentiles of the distribution of SPPB slope and SPPB five years prior to death relative to the 80^th^ percentile. Plots of the RR curves were created to visualize risk relative to the 80^th^ percentile of the SPPB slope and predicted SPPB five years prior to death distributions for a continuous range of values. *P*-values were computed based on Wald tests for estimated relative risk at the 20^th^, 40^th^, and 60^th^ percentiles of SPPB slope or predicted SPPB five years prior to death relative to the 80th percentile.

Statistical significance was evaluated using alpha = 0.05. All statistical analyses were conducted using R 2.15.1.

## Results

### Estimation of SPPB trajectories

**Fig. 2:**
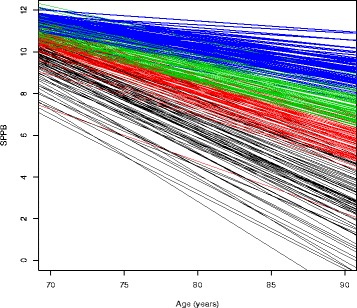
Estimated subject-specific SPPB trajectories^a^ for 305 autopsied ACT participants. ^a^Lines are color coded by quartile of slope. Black = >75^th^ percentile, Red = 50^th^ – 75^th^ percentile, Green = 25^th^ – 50^th^ percentile, Blue = <25^th^ percentile. 75^th^ percentile = -0.148, 50^th^ percentile = -0.196, 25^th^ percentile = -0.256

Among the 3174 participants included in the estimation of physical performance trajectories, the mean number of SPPB measures was 4.2 (standard deviation 1.9), and the range was 2 to 9 measures (23% had 2, 22% had 3, and 55% had 4 or more measurements of SPPB). Estimated SPPB trajectories revealed substantial variation in change in physical performance with age. Compared to the quartile with the steepest decline (SPPB slope < −0.256 per year) shown in black (Fig. 2), the quartile of decedents with the least decline (SPPB slope > −0.148 per year), had higher SPPB scores at baseline on average, had a higher proportion of men (57.9%), had higher average educational attainment, had a higher proportion who regularly exercised, were older on average, and had faster gait speeds at baseline on average (Table 1). There were no apparent differences in classification of dementia or AD at last ACT study visit by quartile of SPPB slope. For comparison, characteristics of the cohort of 2813 participants, stratified by estimated slope of the SPPB trajectory, are provided in Table 5 in Appendix. Although not identical to the associations observed among decedents, many of the patterns described above are similar in the larger cohort.

**Table 1:** Characteristics of ACT autopsy cohort at last study visit by estimated SPPB slope quartiles^a^

	SPPB >75^th^ percentile slope (slower decline) (*N* = 76)	SPPB 50th - 75^th^ percentile slope (*N* = 76)	SPPB 25th - 50^th^ percentile slope (N = 76)	SPPB <25^th^ percentile slope (faster decline) (*N* = 77)
	N (%)	N (%)	N (%)	N (%)
Cohort				
Original cohort	62 (81.6)	66 (86.8)	64 (84.2)	70 (90.9)
Expansion cohort	14 (18.4)	9 (11.8)	12 (15.8)	6 (7.8)
Replacement cohort	0 (0.0)	1 (1.3)	0 (0.0)	1 (1.3)
Sex				
Male	44 (57.9)	38 (50.0)	33 (43.4)	25 (32.5)
Female	32 (42.1)	38 (50.0)	43 (56.6)	52 (67.5)
Age at baseline				
< 70 years	0 (0.0)	4 (5.3)	4 (5.3)	3 (3.9)
70–74 years	5 (6.6)	18 (23.7)	12 (15.8)	12 (15.6)
75–79 years	21 (27.6)	10 (13.2)	16 (21.1)	28 (36.4)
80–84 years	27 (35.5)	21 (27.6)	22 (28.9)	23 (29.9)
85+ years	23 (30.3)	23 (30.3)	22 (28.9)	11 (14.3)
BMI at last ACT study visit				
Underweight (<18.5)	3 (4.0)	0 (0.0)	1 (1.4)	1 (1.3)
Normal weight (18.5–24.99)	34 (45.3)	39 (54.2)	31 (42.5)	23 (30.7)
Overweight (25–29.99)	33 (44.0)	25 (34.7)	25 (34.2)	34 (45.3)
Obese (30+)	5 (6.7)	8 (11.1)	16 (21.9)	17 (22.7)
Dementia				
No dementia	44 (57.9)	39 (51.3)	43 (56.6)	37 (48.1)
Dementia	32 (42.1)	37 (48.7)	33 (43.4)	40 (51.9)
Possible or probable AD				
No AD	47 (61.8)	51 (67.1)	51 (67.1)	46 (59.7)
AD	29 (38.2)	25 (32.9)	25 (32.9)	31 (40.3)
Education				
< 12 years	3 (3.9)	15 (19.7)	8 (10.5)	7 (9.1)
12–15 years	37 (48.7)	33 (43.4)	40 (52.6)	47 (61.0)
16+ years	36 (47.4)	28 (36.8)	28 (36.8)	23 (29.9)
Smoking at last visit				
Never	30 (39.5)	32 (42.1)	31 (40.8)	27 (35.1)
Former	42 (55.3)	40 (52.6)	40 (52.6)	44 (57.1)
Current	4 (5.3)	4 (5.3)	5 (6.6)	6 (7.8)
Regular exercise at last visit				
No	21 (28.0)	21 (27.6)	36 (48.0)	36 (47.4)
Yes	54 (72.0)	55 (72.4)	39 (52.0)	40 (52.6)
SPPB: gait speed^a^ component (0–4) at baseline				
1	1 (1.3)	4 (5.3)	9 (11.8)	15 (19.5)
2	1 (1.3)	10 (13.2)	8 (10.5)	14 (18.2)
3	28 (36.8)	32 (42.1)	34 (44.7)	34 (44.2)
4	46 (60.5)	30 (39.5)	25 (32.9)	14 (18.2)
Grip strength^b^ at baseline				
0	2 (2.6)	0 (0.0)	0 (0.0)	0 (0.0)
1	5 (6.6)	10 (13.2)	14 (18.4)	6 (7.8)
2	15 (19.7)	17 (22.4)	14 (18.4)	28 (36.4)
3	39 (51.3)	31 (40.8)	30 (39.5)	33 (42.9)
4	15 (19.7)	18 (23.7)	18 (23.7)	10 (13.0)

^a^SPPB slope percentile values: 75th percentile = −0.148, 50th percentile = −0.196, 25th percentile = −0.256

^b^Gait speed and grip strength are rated on a 0–4 scale with 0 indicating unable to perform test, and higher scores indicating greater function (faster gait speed and stronger grip strength). Scales are defined based on sex-specific cutpoints: grip strength (male: <25 kg, 25 to <30, 30 to <40, and ≥40; female: <15 kg, 15 to <20, 20 to <25, and ≥25); gait speed based on time to walk 10-ft (men: ≥ 5 s, 4.5 s, 3.5 to 4 s, and ≤3 s; women: ≥ 5.5 s, 4.5 to 5 s, 3.5 to 4 s, and ≤3 s)

### Associations of SPPB trajectories with Neuropathologic findings at autopsy

Unadjusted distributions of CERAD (neuritic plaques), Braak (neurofibrillary tangles) and amyloid angiopathy neuropathology classifications revealed no orderly pattern across quartiles of SPPB slope, while microinfarcts and cystic infarcts appeared more common among decedents with the fastest SPPB declines (Table 2). After adjustment for sex, age, BMI and education, no statistically significant associations were seen for plaques or tangles, but relative risks were in the direction of lower risk of these neuropathologies among autopsied decedents who had steeper declines in SPPB prior to death (Table 3). Risk of amyloid angiopathy was significantly lower among decedents with the fastest declines: compared to the 80^th^ percentile SPPB slope (least decline), the relative risk was 0.81 (95% CI 0.63–1.01; *p* = 0.09) for the 60^th^ percentile of SPPB slope, 0.70 (95% CI, 0.47–0.99, *p* = 0.07) for the 40^th^ percentile of SPPB slope and 0.58 (95% CI, 0.34–0.92, *p* = 0.02) for the 20^th^ percentile of SPPB slope (Table 3; Fig. 2).

**Table 2:** Unadjusted proportions for binary neuropathology outcomes according to quartiles^a^ of SPPB slope

	SPPB >75^th^ percentile slope (slower decline) (*N* = 76)	SPPB 50th - 75^th^ percentile slope (*N* = 76)	SPPB 25th - 50^th^ percentile slope (*N* = 76)	SPPB <25^th^ percentile slope (faster decline) (*N* = 77)
	*N* (%)	*N* (%)	*N* (%)	*N* (%)
CERAD (plaques)				
Intermediate/frequent	40 (52.6)	31 (40.8)	35 (46.1)	41 (53.2)
None/sparse	36 (47.4)	45 (59.2)	41 (53.9)	36 (46.8)
Braak (tangles)				
V-VI	29 (38.2)	17 (22.4)	25 (32.9)	25 (32.5)
0-IV	47 (61.8)	59 (77.6)	51 (67.1)	52 (67.5)
Amyloid angiopathy				
Mild, moderate, or severe	25 (32.9)	23 (30.7)	18 (24.0)	22 (28.6)
None	51 (67.1)	52 (69.3)	57 (76.0)	55 (71.4)
Microinfarcts				
> 2 cerebral or deep	8 (10.5)	5 (6.6)	9 (11.8)	21 (27.3)
< = 2 cerebral or deep	68 (89.5)	71 (93.4)	67 (88.2)	56 (72.7)
Cystic infarcts				
1 or more	14 (18.9)	22 (30.1)	25 (34.2)	24 (31.2)
None	60 (81.1)	51 (69.9)	48 (65.8)	53 (68.8)
Atherosclerosis				
2–3	45 (62.5)	44 (58.7)	35 (50.0)	48 (63.2)
0–1	27 (37.5)	31 (41.3)	35 (50.0)	28 (36.8)
Cortical Lewy bodies				
1	3 (3.9)	6 (8.0)	5 (6.6)	3 (3.9)
0	73 (96.1)	69 (92.0)	71 (93.4)	74 (96.1)

^a^SPPB slope percentile values: 75^th^ percentile = −0.148, 50^th^ percentile = −0.196, 25th percentile = −0.256

**Table 3:** Relative risks for neuropathology outcomes by rate of change in SPPB

	Rate of change of SPPB	Risk of neuropath outcomes		
		RR	95%CI	P
CERAD (plaques) (intermediate/frequent)				
80^th^ percentile (slower decline)	−0.124	Ref	–	–
60	−0.163	0.88	(0.74, 1.05)	0.14
40	−0.197	0.82	(0.62, 1.07)	0.158
20^th^ percentile (faster decline)	−0.251	0.80	(0.57, 1.14)	0.212
Braak (tangles) (V-VI)				
80^th^ percentile (slower decline)	−0.124	Ref	–	–
60	−0.163	0.81	(0.65, 1.07)	0.12
40	−0.197	0.72	(0.51, 1.09)	0.11
20^th^ percentile (faster decline)	−0.251	0.66	(0.41, 1.02)	0.08
Amyloid angiopathy (mild, moderate, or severe)				
80^th^ percentile (slower decline)	−0.124	Ref	–	–
60	−0.163	0.81	(0.63, 1.01)	0.09
40	−0.197	0.70	(0.47, 0.99)	0.07
20^th^ percentile (faster decline)	−0.251	0.58	(0.34, 0.92)	0.02
Microinfarcts (>2 cerebral or deep)				
80^th^ percentile (slower decline)	−0.124	Ref	–	–
60	−0.163	1.06	(0.69, 1.61)	0.80
40	−0.197	1.15	(0.57, 2.22)	0.72
20^th^ percentile (faster decline)	−0.251	1.45	(0.55, 3.61)	0.41
Cystic infarcts (1 or more)				
80^th^ percentile (slower decline)	−0.124	Ref	–	–
60	−0.163	1.35	(1.00, 1.82)	0.04
40	−0.197	1.58	(0.97, 2.50)	0.06
20^th^ percentile (faster decline)	−0.251	1.64	(0.84, 2.92)	0.11
Atherosclerosis (2–3)				
80^th^ percentile (slower decline)	−0.124	Ref	–	–
60	−0.163	0.96	(0.85, 1.11)	0.51
40	−0.197	0.94	(0.79, 1.21)	0.59
20^th^ percentile (faster decline)	−0.251	0.98	(0.79, 1.35)	0.88

Relative risks are computed for 20th, 40th, and 60th percentiles of slope relative to 80th percentile of slope. Adjusted for sex, age, BMI, and education

**Fig. 3:**
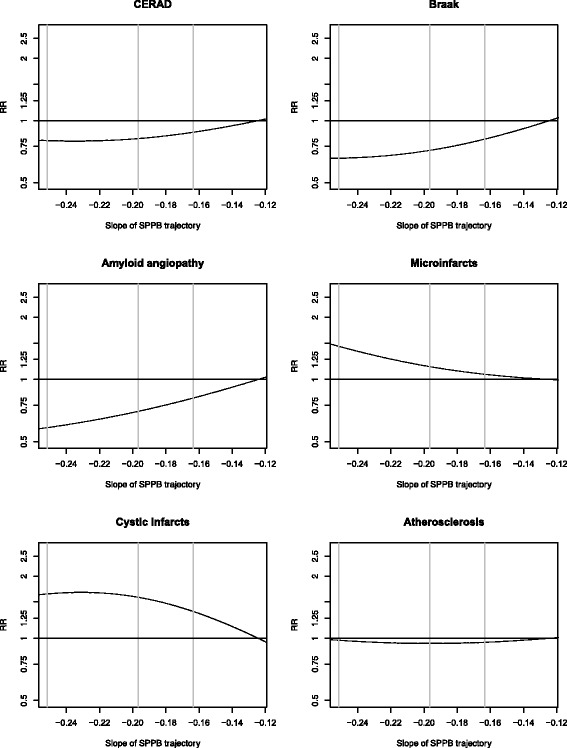
Relative risks^a^ relating slope of SPPB with neuropathology outcomes based on natural cubic splines. ^a^Model includes slope estimated via weighted GEE adjusted for sex, age, BMI, and education computed relative to the 80^th^ percentile of SPPB slope. Vertical lines indicate 20^th^, 40^th^, and 60^th^ percentiles of SPPB slope

There were no statistically significant associations between risk of microinfarcts, cystic infarcts or atherosclerosis neuropathology outcomes and slope of SPPB decline. However, the pattern of results suggested higher relative risks for microinfarcts and cystic infarcts among the fastest decliners (Table 3; Fig. 3). As shown in the natural cubic spline plots of the relative risks across the range of SPPB slopes (Fig. 3), this pattern of results is consistent with either no association or lower risk of AD pathology (CERAD, Braak, amyloid angiopathy) and higher risk of vascular (microinfarcts and cystic infarcts) neuropathology among decedents with faster rates of SPPB decline.

**Fig. 4:**
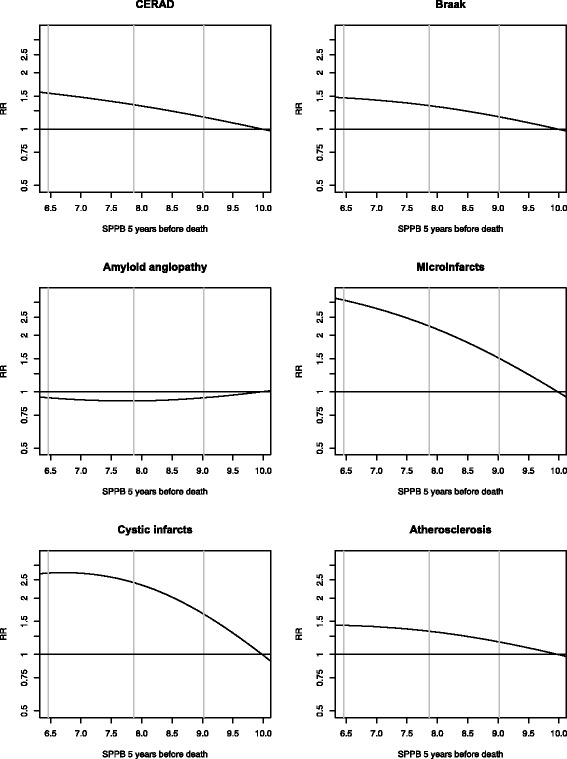
Relative risks^a^ relating estimated SPPB 5 years before death to neuropathology outcomes. ^a^Model includes SPPB 5 years before death estimated via weighted GEE adjusted for sex, age, BMI, and education computed relative to the 80^th^ percentile of SPPB slope. Vertical lines indicate 20^th^, 40^th^, and 60^th^ percentiles of SPPB slope

When we examined the predicted SPPB scores 5 years prior to death in relation to the neuropathology outcomes, we observed higher risks for lower SPPB scores for all neuropathology outcomes except for amyloid angiopathy; the highest relative risks were observed for microinfarcts (RRs = 2.24 and 3.08 for the 40th and 20th vs. 80th percentiles of SPPB scores, respectively) and cystic infarcts (RRs = 2.42 and 2.72 for the 40th and 20th vs. 80th percentiles of SPPB scores, respectively; Table 4). The relative risks had wide 95% confidence intervals and were only statistically significant for cystic infarcts. The natural cubic spline plots for these relative risks illustrate the increased risk of neuropathology outcomes associated with low predicted SPPB scores 5 years prior to death (Fig. 4).

**Table 4:** Relative risks^a^ for neuropathology outcomes for percentiles of estimated SPPB 5 years before death

	SPPB 5 years before death	Risk of neuropath outcomes		
		RR^b^	95%CI	P
CERAD (plaques) (intermediate/frequent)				
80^th^ percentile (higher SPPB)	9.98	Ref	–	–
60	9.02	1.16	(0.94, 1.51)	0.24
40	7.86	1.35	(0.94, 2.17)	0.15
20^th^ percentile (lower SPPB)	6.46	1.55	(1.02, 2.73)	0.06
Braak (tangles) (V-VI)				
80^th^ percentile (higher SPPB)	9.98	Ref	–	–
60	9.02	1.16	(0.87, 1.55)	0.31
40	7.86	1.33	(0.80, 2.24)	0.29
20^th^ percentile (lower SPPB)	6.46	1.47	(0.81, 2.80)	0.24
Amyloid angiopathy (mild, moderate, or severe)				
80^th^ percentile (higher SPPB)	9.98	Ref	–	–
60	9.02	0.93	(0.70, 1.24)	0.63
40	7.86	0.89	(0.52, 1.48)	0.66
20th percentile (lower SPPB)	6.46	0.93	(0.51, 1.67)	0.78
Microinfarcts (>2 cerebral or deep)				
80^th^ percentile (higher SPPB)	9.98	Ref	–	–
60	9.02	1.50	(0.88, 2.57)	0.13
40	7.86	2.24	(0.84, 6.01)	0.10
20^th^ percentile (lower SPPB)	6.46	3.08	(0.93, 10.07)	0.07
Cystic infarcts (1 or more)				
80^th^ percentile (higher SPPB)	9.98	Ref	–	–
60	9.02	1.64	(1.17, 2.32)	0.002
40	7.86	2.42	(1.37, 4.51)	0.002
20^th^ percentile (lower SPPB)	6.46	2.72	(1.45, 5.57)	0.005
Atherosclerosis (2–3)				
80^th^ percentile (higher SPPB)	9.98	Ref	–	–
60	9.02	1.17	(0.95, 1.39)	0.11
40	7.86	1.33	(0.93, 1.80)	0.10
20^th^ percentile (lower SPPB)	6.46	1.43	(0.96, 1.99)	0.08

^a^Estimated via weighted GEE

^b^Relative risks are computed for 20^th^, 40^th^, and 60^th^ percentiles relative to 80th percentiles from modified Poisson regression models adjusted for sex, age, BMI, and education

## Discussion

Based on up to 16 years of follow-up and 2–9 measurements of SPPB among 3174 men and women aged 65 and older, we observed considerable variability in the trajectories of physical function in the ACT cohort. Some older adults maintained function almost entirely, while others lost function at a very rapid rate. Among decedents for whom we had autopsy neuropathology data, there was no evidence of increased risk in AD pathology as assessed by CERAD level and Braak stage associated with more rapid decline in SPPB scores. In fact, we found suggestions that the risk of plaques, tangles and amyloid angiopathy were in the opposite direction, significantly so for amyloid angiopathy, suggesting a somewhat lower rate of these pathologies in people with relatively rapidly declining SPPB scores. In contrast, relative risks for the vascular neuropathologies of microinfarcts and cystic infarcts were higher among people with relatively rapidly declining SPPB scores, though not significantly elevated for any type of lesion. Relative risks for all of the pathological findings were in the direction of increased risk when we examined estimated SPPB scores 5 years prior to death. The relative risks were highest for microinfarcts and cystic infarcts suggesting a 2–3 fold increased risk of these neuropathologies among decedents with the lowest predicted SPPB score. However, statistically significant associations were observed only for cystic infarcts.

Evidence has accumulated over the last decade that physical and cognitive function are linked. Numerous associations have been observed in prospective studies that examined each domain of functional status in relation to the future trajectory of the other [27]. Epidemiologic studies have also established associations between frailty and incidence of mild cognitive impairment [28], Alzheimer’s Disease [29], and dementia [9, 10, 30]. In our previous study on this topic in the ACT cohort, frailty was associated with higher risk for dementia (HR = 1.2), but the association was solely due to an increased risk of non-AD dementia (HR = 2.6), and was not apparent for AD dementia [10]. Similarly, in the Italian Longitudinal Study of Aging, Solfrizzi [9] and colleagues found that frailty was associated with a decreased risk of AD (HR = 0.62), but an increased risk of vascular and other dementias (HRs = 2.7). These findings align well with this report in which risks of micro- and cystic infarcts were higher among those with the fastest rates of decline in SPPB score, but no increased risks of plaques or tangles were observed.

In the present study, we also observed increased risks of both AD and vascular neuropathologies with lower SPPB scores estimated 5 years prior to death. Although highest for micro- and cystic infarcts and statistically significant only for cystic infarcts, relative risks were in the direction of increased risk for lower SPPB scores estimated 5 years before death. Among 791 older adults from the Religious Orders Study and the Rush Memory and Aging Project, a faster rate of change in frailty in the 6 years prior to death was associated with many AD and vascular pathology findings, although together the brain lesions explained only 8% of the variation in frailty progression [7]. When considered together with our results from the estimated SPPB five years prior to death, these findings suggest that neuropathological findings of any type are associated with lower levels of physical performance in the years just prior to death. Our findings on slopes suggest that higher rates of functional decline may be especially likely for people with vascular pathology. Such observations may reflect some terminal decline that occurs simultaneously in many functional domains, but is not specific to a particular brain disease. A recent review of frailty and cognitive impairment concluded that the observed reciprocal relationships suggest “that cognition and frailty interact within a cycle of decline associated with ageing” [31].

The common element between the constructs of frailty and SPPB is gait speed as measured by timed gait. In a recent review of 27 studies examining gait speed in relation to structural brain findings on MRI, numerous associations between gait speed and white and gray matter volume have been observed, but the evidence base is quite mixed and inconsistent [5]. For example, in the Cardiovascular Health Study, slower gait speed and faster decline in gait speed were associated with ventricular enlargement and white matter hyperintensities on MR [32, 33], but confirmatory evidence is lacking. Strong cross-sectional associations between SPPB and severity of age-related white matter changes have also been observed in the Leukoaraiosis and Disability Study (LADIS) [34]. Studies of global brain networks are being done in an effort to understand the impact of functional changes in the brain in relation to mobility changes in older adults. A recent, small study found striking differences among older adults with low and high SPPB scores in connectivity in the somatomotor cortex [35]. While much remains to be learned about how cognitive aging and subclinical cognitive diseases affect trajectories of physical function in the last decades of life, the emerging evidence suggests a definite role of brain aging and disease in this process.

Although the evidence above supports the biologic plausibility of a role for brain aging and disease in physical function losses with aging, there are many alternative explanations. An association of declining physical function with vascular neuropathological findings could be explained by an impact of brain function on motor ability [4], or the associations could be explained by shared, underlying vascular pathology in the microvasculature and common impacts of risk factors that contribute to microvascular disease such as inflammation and oxidative stress. The different associations between trajectories of SPPB and AD vs. vascular neuropathology point to different etiologies, and future longitudinal studies should examine whether cardiovascular disease and risk factors mediate this association. Our analyses adjust for sex, age, education and BMI, but we did not measure biomarker levels in the ACT cohort.

Strengths of our study include the evaluation of a population-based autopsy cohort with well characterized health status, health behaviors, physical function and cognitive status over time, as well as state-of-the-art methods for ascertaining brain neuropathology. We accounted for selection factors related to inclusion in the autopsy cohort by applying inverse-probability weights to improve generalizability of these results to the entire population-based cohort. We were able to study SPPB trajectories using up to 9 measurements; 75% of study participants had at least 3 SPPB measurements. Follow-up time in the autopsy cohort is shorter than the entire cohort, because participants are necessarily censored at time of death. In addition, in the ACT cohort, once a participant exhibits decline in global cognition as measured by the CASI score, they are evaluated for dementia using standardized protocols. If dementia is identified, the participant has a single follow-up visit using the same protocol, and if dementia is confirmed, they are followed only by telephone, without further measures of the SPPB, which requires an in-person visit. Thus, the SPPB measurements used in this analysis were all measured before any participants had a clinical diagnosis of dementia. This study was limited by the relatively small sample size of autopsied ACT decedents. We did not have measurements of long-tract white matter disease to include in this analysis and numbers of autopsies with corticol Lewy body indices were too small to produce meaningful results. Future studies should examine an expanded array of brain neuropathologies in relation to physical function prior to death.

## Conclusion

Cystic infarcts and microinfarcts, but not neuropathology findings of Alzheimer’s disease, were related to physical performance levels five years before death. The rate of physical performance decline was not associated with either vascular or Alzheimer’s disease brain neuropathology. These findings add to accumulating evidence that brain aging and disease contribute to physical function levels in older people in the years preceding death. The findings support identifying modifiable risk factors associated with vascular neuropathological findings and testing whether intervening on these risk factors can preserve physical function and reduce brain neuropathology in older adults.

## Appendix

**Table 5:** Characteristics of all ACT participants at last study visit by quartiles of SPPB slope

	SPPB >75th percentile slope (slower decline) (*N* = 872)	SPPB 50th - 75th percentile slope (*N* = 803)	SPPB 25th - 50th percentile slope (*N* = 610)	SPPB <25th percentile slope (faster decline) (*N* = 528)
	*N* (%)	*N* (%)	*N* (%)	*N* (%)
Cohort				
Original cohort	574 (65.8)	463 (57.7)	415 (68.0)	415 (78.6)
Expansion cohort	202 (23.2)	156 (19.4)	123 (20.2)	88 (16.7)
Replacement cohort	96 (11.0)	184 (22.9)	72 (11.8)	25 (4.7)
Sex				
Male	410 (47.0)	371 (46.2)	229 (37.5)	164 (31.1)
Female	462 (53.0)	432 (53.8)	381 (62.5)	364 (68.9)
Age at baseline				
< 70 years	33 (3.8)	120 (14.9)	48 (7.9)	26 (4.9)
70–74 years	143 (16.4)	255 (31.8)	149 (24.4)	123 (23.3)
75–79 years	241 (27.6)	189 (23.5)	149 (24.4)	172 (32.6)
80–84 years	246 (28.2)	133 (16.6)	157 (25.7)	146 (27.7)
85+ years	209 (24.0)	106 (13.2)	107 (17.5)	61 (11.6)
BMI at last ACT study visit				
Underweight (<18.5)	15 (1.7)	12 (1.5)	6 (1.0)	2 (0.4)
Normal weight (18.5–24.99)	354 (41.1)	283 (35.6)	182 (30.3)	142 (27.4)
Overweight (25–29.99)	378 (43.9)	332 (41.8)	241 (40.1)	202 (39.0)
Obese (30+)	114 (13.2)	168 (21.1)	172 (28.6)	172 (33.2)
Dementia				
No dementia	698 (80.0)	638 (79.5)	423 (69.3)	361 (68.4)
Dementia	174 (20.0)	165 (20.5)	187 (30.7)	167 (31.6)
Possible or probable AD				
No AD	720 (82.6)	672 (83.7)	461 (75.6)	398 (75.4)
AD	152 (17.4)	131 (16.3)	149 (24.4)	130 (24.6)
Education				
< 12 years	89 (10.2)	71 (8.8)	69 (11.3)	67 (12.7)
12–15 years	395 (45.3)	366 (45.6)	316 (51.8)	315 (59.7)
16+ years	388 (44.5)	366 (45.6)	225 (36.9)	146 (27.7)
Smoking at last visit				
Never	427 (49.5)	378 (47.3)	300 (49.5)	241 (45.9)
Former	419 (48.6)	390 (48.8)	270 (44.6)	260 (49.5)
Current	17 (2.0)	31 (3.9)	36 (5.9)	24 (4.6)
Regular exercise at last visit				
No	222 (25.7)	262 (32.8)	234 (38.5)	258 (49.2)
Yes	643 (74.3)	537 (67.2)	374 (61.5)	266 (50.8)
SPPB: gait speed^a^ component (0–4) at baseline				
1	14 (1.6)	35 (4.4)	42 (6.9)	102 (19.3)
2	31 (3.6)	60 (7.5)	59 (9.7)	91 (17.2)
3	260 (29.8)	267 (33.3)	251 (41.1)	194 (36.7)
4	567 (65.0)	441 (54.9)	258 (42.3)	141 (26.7)
Grip strength^a^ at baseline				
0	9 (1.0)	8 (1.0)	6 (1.0)	3 (0.6)
1	40 (4.6)	59 (7.3)	66 (10.8)	67 (12.7)
2	145 (16.6)	152 (18.9)	137 (22.5)	145 (27.6)
3	382 (43.8)	331 (41.2)	249 (40.8)	209 (39.7)
4	296 (33.9)	253 (31.5)	152 (24.9)	102 (19.4)

^a^ Quartiles defined based on ACT autopsy subcohort

^b^Gait speed and grip strength are rated on a 0–4 scale with 0 indicating unable to perform test, and higher scores indicating greater function (faster gait speed and stronger grip strength). Scales are defined based on sex-specific cutpoints: grip strength (men: <25 kg, 25 to <30, 30 to <40, and ≥40; women: <15 kg, 15 to <20, 20 to <25, and ≥25); gait speed based on time to walk 10-ft (men: ≥ 5 s, 4.5 s, 3.5 to 4 s, and ≤3 s; women: ≥ 5.5 s, 4.5 to 5 s, 3.5 to 4 s, and ≤3 s)

## Abbreviations

ACT: Adult Changes in Thought
AD: Alzheimer’s Disease
BMI: Body Mass Index
CERAD: Consortium to Establish a Registry for Alzheimer’s Disease
GEE: Generalized Estimating Eqs.
GH: Group Health
IPW: Inverse Probability Weights
RR: Relative Risk
SPPB: Short Physical Performance Battery
